# Core competencies in applied infectious disease epidemiology: a framework for countries in Europe

**DOI:** 10.2807/1560-7917.ES.2023.28.6.2200517

**Published:** 2023-02-09

**Authors:** Amelie Plymoth, Mary B Codd, Julia Barry, Adrian Boncan, Arnold Bosman, Karl F Conyard, Katarzyna Czabanowska, Nadav Davidovitch, Rodrigo Filipe, Lorena Gonzalez, Lore Leighton, John Middleton, Michael Ndirangu, Robert Otok, John Reid, Ralf Reintjes, Darren Shickle, Shiraz Syed, Patrick Wall, Jeanine Pommier

**Affiliations:** 1 European Centre for Disease Prevention and Control (ECDC), Stockholm, Sweden; 2 University College Dublin, Dublin, Ireland; 3 The Association of Schools of Public Health in the European Region (ASPHER), Brussels, Belgium; 4 Transmissible BV, Utrecht, The Netherlands; 5 Care and Public Health Research Institute (CAPHRI), Maastricht University, The Netherlands; 6 Ben Gurion University of the Negev, Be'er Sheva, Israel; 7 University of Wolverhampton, United Kingdom; 8 University of Chester, United Kingdom; 9 Hamburg University of Applied Sciences, Hamburg, Germany; 10 Tampere University, Tampere, Finland; 11 University of Leeds, Leeds, United Kingdom

**Keywords:** core competencies, infectious diseases, epidemiology, public health, self-assessment, prevention, control, ASPHER, European Union, ECDC, workforce, public health expert, framework

## Abstract

In 2009, the European Centre for Disease Prevention and Control (ECDC) developed a competency framework to support European Union countries and the European Commission in ensuring a competent public health workforce for Europe. The coronavirus disease (COVID-19) pandemic emphasised the importance of harmonised public health strategies and competencies across international boundaries, specifically for infectious diseases. This perspective presents the process to update the competency framework for applied infectious disease epidemiology, highlighting ECDC’s efforts to support countries with using the framework. ECDC commissioned the Association of Schools of Public Health in the European Region (ASPHER) to update the framework through publication and dissemination of a technical report and a self-assessment tool linked to training resources. A mixed methods approach to gather input from experts in relevant specialities included qualitative interviews with 42 experts, workshops with ECDC Technical Advisory Group and an online survey of 212 public health professionals across Europe and beyond. Modifications resulted in 157 core competencies in 23 domains, each mapping to one of six subject areas of importance in applied infectious disease epidemiology. The framework serves as a basis to update the curriculum of the ECDC Fellowship programme with two alternative paths: intervention epidemiology or public health microbiology.

## Background

The European Centre for Disease Prevention and Control (ECDC) supports the European Union (EU) countries and the European Commission to ensure a competent public health workforce for Europe. Core competencies for public health epidemiologists working in communicable disease surveillance, outbreak investigation and emergency response in the EU were published by ECDC in 2009 [1]. In this report it was recognised that competencies “*will be updated periodically, in collaboration with its intended users (public health institutes in the EU, training programmes, etc.)*” [1]*.* Twelve years later, it was considered important to update the core competencies in applied infectious disease epidemiology to: (i) reflect new approaches to public health practice [2-13]; (ii) support the development of a workforce with the necessary knowledge, skills and abilities to translate policy, theory, and research into action, in light of recent and current infectious disease epidemics and pandemics [14-17]; (iii) share a common vision of the specific knowledge and skills required for essential and effective education and training to develop and strengthen the public health workforce in the 21st century. This is in line with the World Health Organization (WHO) Essential Public Health Operation 7 (EPHO 7), which aims at *‘assuring a sufficient and competent public health workforce*’. Investment in, and development of, a public health workforce is an essential prerequisite for adequate delivery and implementation of public health services and activities [18]. This is also in accordance with the European Union of Medical Specialists (UEMS), which monitors and evaluates medical speciality training in all European countries including clinical (medical) microbiology, infectious diseases and public health medicine [19].

The coronavirus disease (COVID-19) pandemic emphasised the importance of harmonised public health strategies and competencies across international boundaries, specifically in relation to infectious diseases.

We document the process to update the competency framework, commissioned by ECDC and carried out by the Association of Schools of Public Health in the European Region (ASPHER). Based on this work, ECDC is supporting countries through several different mechanisms to use the framework.

## Purpose, scope and approach

The purpose of this work was to update the competency framework’s previously defined core competencies in applied infectious disease epidemiology to reflect changes and developments in the field, and to support the training and assessment of public health professionals of the future. To facilitate this, a self-assessment tool kit based on the updated core competencies was developed so that professionals can assess their competency level in each domain.

The main target audience for the framework is mid-career professionals, defined as those having approximately 5 years experience of professional practice relevant to applied infectious diseases epidemiology and an advanced degree with a specialisation in public health, epidemiology, or other related field (e.g. immunology, microbiology, parasitology, vector control, environmental health, One Health). Job titles may include field epidemiologist, infectious diseases epidemiologist or public health specialist focusing on infectious disease investigation and management, but titles may vary across countries.

The Association of Schools of Public Health in the European Region was commissioned by ECDC in 2020 to review relevant competency frameworks and published literature to identify new and important competencies for applied infectious disease epidemiology. The international working group comprised members with expertise in epidemiology, biostatistics, infectious disease, public health medicine, infodemiology, veterinary medicine, education, governance, business administration, One Health, outbreak response, sociology, surveillance, disaster management, leadership and workforce development. All members were actively engaged in teaching and public health responses to COVID-19 in their respective countries.

A preliminary set of core competencies was developed as a draft document and used as the basis for further consultation. A mixed-methods approach was used to gather input from experts in relevant specialities. This included structured qualitative interviews with 42 nominated experts and an online workshop with representatives of the ECDC Technical Advisory Group (TAG). The Advisory Group consisted of approximately 16 participants including ECDC experts and European partners from the policy, practice, and academic sectors with experience in applied epidemiology and/or on the development of competencies. The scope of the advisory group was to provide nonbinding strategic advice to the project team and to guide the definition of boundaries and the content of the competencies. The members provided expertise through discussion meetings, provision of articles and other resources useful to the project, participation in interviews, and reviews of intermediary drafts of the framework.

As a result of this input, a revised set of core competencies was used as the basis of an online survey with a wider professional audience worldwide. Valuable feedback from 212 responders resulted in further revisions, which, after further review by 10 expert reviewers in the commissioned team, was finalised.

## Updated competency framework

The updated competency framework [20] resulting from this work comprises 157 core competencies in 23 domains, each of which are now mapped to one of six subject areas of importance in applied infectious disease epidemiology. The subject areas are displayed in the Box.

Box **Subject areas of importance in the applied infectious disease epidemiology core competencies framework, European Centre for Disease Prevention and Control, 2021**

**Area A. Essential methods for applied infectious diseases epidemiology**. A competent mid-career professional in applied infectious disease epidemiology should have a thorough understanding of epidemiology, research methods, data management and biostatistics. They should be skilled in applying research methods, synthesising knowledge and interpreting disease surveillance and investigation data.

**Area B. Preparedness, surveillance and response to infectious disease outbreaks.** The public health response to any infectious disease outbreak, epidemic or pandemic requires a level of preparedness, active systems of surveillance and swift public health responses across many specialities and domains.

**Area C. Communication and advocacy.** Clear communication of policies, strategies and public health messages, strong engagement and advocacy skills and modern communication and advocacy techniques designed to reach selected groups in organisations and communities are critical to the public health response to infectious disease outbreaks.

**Area D. Practice of infectious disease epidemiology.** Appropriate and professional action on case definition, case identification, testing, diagnosis, contact management, isolation, support and treatment are essential components of a health system’s response to infectious disease threats.

**Area E. Contextual influences on infectious disease management.** Contextual and systemic influences on infectious disease management include the impact of the political system of the country or region, the organisation and structure of health services and their delivery, how it influences health equity especially related to vulnerable groups and the understanding of socioeconomic and sociocultural contexts of the country or region. These may impact access to, and the delivery of, services relevant to infectious diseases.

**Area F. Leadership and management.** Competencies in leadership and management are required to develop and implement policies on management of infectious disease outbreaks, epidemics and pandemics. Policy implementation is key in any organisational framework to guide the actions of individuals, promote consistency and ensure standardisation across the board.

The updated framework includes new and reinforced emergency preparedness and response domains linked to Internal Health Regulation (IHR) requirements [21]. It strengthens the importance of evaluation approaches, as well as the comprehensive knowledge of the existing legislative frameworks specific to surveillance and reporting of diseases. Competencies needed to address emerging public health issues related to infodemic management and vaccine misinformation are also included. Collaboration with specialists in infodemiology provides credibility to disseminating public health information on social media platforms. Other new competencies emphasise the impact of climate change and the increasingly important One Health concept addressing interactions of the human and animal worlds. The importance and harmonisation of competencies linked to collaboration between epidemiologists and numerous stakeholders is also highlighted.

**Figure 1:**
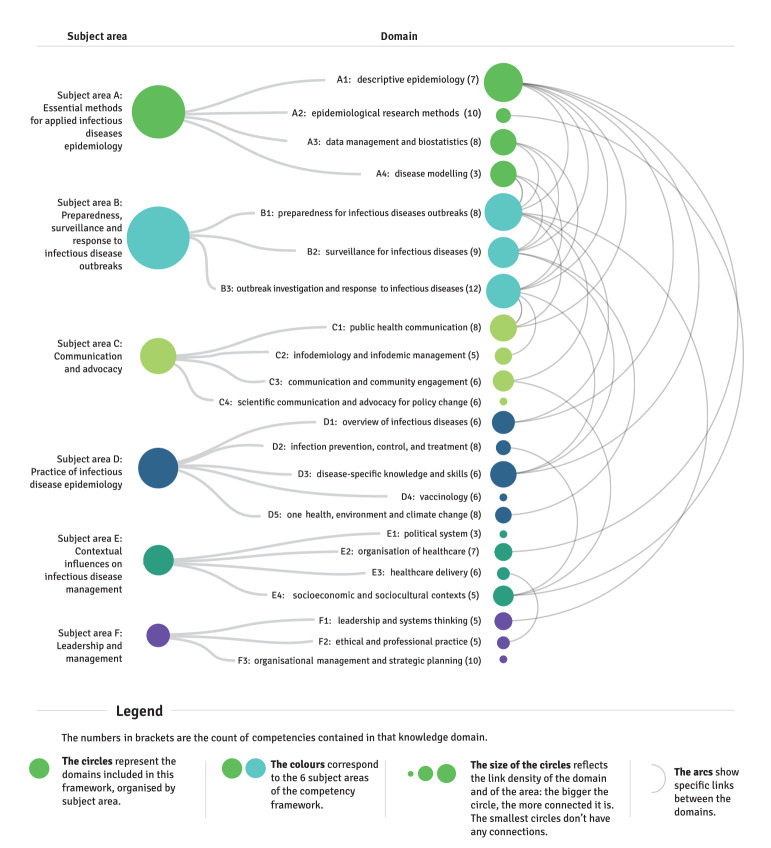
Applied infectious disease epidemiology competency framework: organisation by subject area, domain and cross-referencing of domains, European Centre for Disease Prevention and Control, 2021

Figure 1 provides an overview of the subject areas, domains in each subject area and the number of competencies in each domain. Inevitably, there is overlap between the subject areas and domains of the competency framework; thus, linkages are shown as cross-referencing arcs between domains.

## Resources developed to support countries in using the framework

To facilitate the use of the updated applied infectious disease competency framework, ECDC is implementing different approaches such as a self-assessment toolkit, information meetings, conferences, workshops and scientific communication. The main approach is a self-assessment toolkit based on the updated competency framework. The toolkit, which helps identify an individual’s strongest competencies and areas for improvement, is available in the ECDC Virtual Academy (EVA) [22]. It works entirely offline in the user’s browser enabling them to self-evaluate privately. These data are not accessible by ECDC. Results are saved locally and are printable.

**Figure 2:**
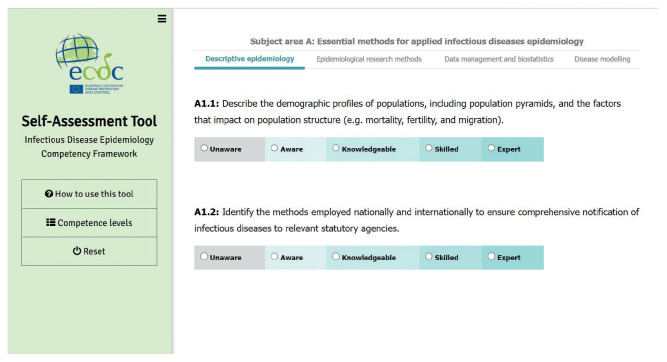
Applied infectious disease epidemiology competency framework self-assessment tool: self-assessed proficiency levels, European Centre for Disease Prevention and Control, 2021

**Figure 3:**
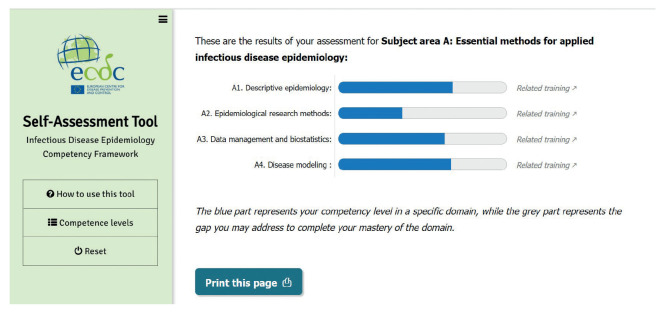
Applied infectious disease epidemiology competency framework self-assessment tool: results of a self-assessment for subject area A, essential methods for applied infectious disease epidemiology, European Centre for Disease Prevention and Control, 2021

For each competency within subject areas and domains, the user can self-assess their level of proficiency from the following range: unaware, aware, knowledgeable, skilled, or expert (Figure 2). The tool also provides a visual report showing the aggregated competency level by domain (Figure 3). Training resources linked to the competencies can support individuals in developing their competencies in defined areas. Once a competency is clicked, related courses and training materials, which are derived from the ECDC Virtual Academy (EVA) and Open WHO platform [23] are accessible. The Open WHO is an interactive, web-based and knowledge-transfer platform offering online courses to improve the response to health emergencies.

A harmonised competency framework facilitates collaboration across international borders and provides common information to professionals who tackle cross-border issues in Europe. To facilitate its use and to further support competencies in the countries, ECDC has translated the original English version of the framework into all EU/European Economic Area (EEA) languages.

## Conclusions

As stated in the background section, the importance of core competencies in applied infectious disease epidemiology has become more evident in the light of recent and current infectious disease epidemics and pandemics. A competent public health workforce is critical to translate public health knowledge and scientific evidence into practice, policy, theory, research, effective action and ultimately outcomes.

This updated competency framework is intended to contribute towards public health workforce capacity building in Europe. It is an important contribution to the future training and education of young professionals in public health, providing a benchmark for quality assurance in the public health response to infectious disease outbreaks, epidemics, and pandemics. Potential uses include curriculum reviews in academic public health programmes, training needs assessments in public health institutions, individual assessments, professional development planning, job descriptions, recruitment strategies and employer support. The framework will also serve as a reference in the ongoing process of updating the curriculum of the ECDC Fellowship programme with the two alternative paths: intervention epidemiology (EPIET) or public health microbiology (EUPHEM).

We believe the competency framework presented provides a thorough picture of what is required of public health professionals working in applied field epidemiology today and what future public health professionals will require. Compared with the ECDC framework 2009, the number of competencies has doubled and have been mapped to six relevant areas of infectious disease. However, new challenges will continue to present themselves requiring updated knowledge and skills. As core competencies continue to evolve, the framework presented will be monitored and updated by ECDC to contribute to the continuous professional development of public health professionals in applied infectious disease epidemiology and related disciplines. It is also noted that many of these competencies can also be applied to non-communicable disease epidemiology.

Hopefully, this updated core competency set will serve as the basis for harmonisation of approaches to the prevention of, and protection from infectious diseases across relevant specialities. It should also act as a catalyst for the development and standardisation of educational programmes in field epidemiology and related specialties. To facilitate its use internationally, the framework has been translated and is available in 26 languages. The ultimate goal is to strengthen competency levels of public health professionals and field epidemiologists, but also others whose remit and specialisation are critical to protecting populations against future infectious disease outbreaks, epidemics and pandemics.
